# Wearable Laser Doppler Flowmetry Sensor: A Feasibility Study with Smoker and Non-Smoker Volunteers

**DOI:** 10.3390/bios10120201

**Published:** 2020-12-07

**Authors:** Mou Saha, Viktor Dremin, Ilya Rafailov, Andrey Dunaev, Sergei Sokolovski, Edik Rafailov

**Affiliations:** 1 Aston Institute of Photonic Technologies, Aston University, Birmingham B4 7ET, UK; s.sokolovsky@aston.ac.uk (S.S.); e.rafailov@aston.ac.uk (E.R.); 2 Research & Development Center of Biomedical Photonics, Orel State University, 302026 Orel, Russia; dunaev@bmecenter.ru; 3 Aston Medical Technology Ltd., Birmingham B7 4BB, UK; i.e.rafailov@outlook.com; 4 Saratov State University, 410012 Saratov, Russia

**Keywords:** wearable laser Doppler flowmetry, blood perfusion, wavelet analysis, smokers

## Abstract

Novel, non-invasive wearable laser Doppler flowmetry (LDF) devices measure real-time blood circulation of the left middle fingertip and the topside of the wrist of the left hand. The LDF signals are simultaneously recorded for fingertip and wrist. The amplitude of blood flow signals and wavelet analysis of the signal are used for the analysis of blood perfusion parameters. The aim of this pilot study is to validate the accuracy of blood circulation measurements recorded by one such non-invasive wearable LDF device for healthy young non-smokers and smokers. This study reveals a higher level of blood perfusion in the non-smoker group compared to the smoker group and vice-versa for the variation of pulse frequency. This result can be useful to assess the sensitivity of the wearable LDF sensor in determining the effect of nicotine for smokers as compared to non-smokers and also the blood microcirculation in smokers with different pathologies.

## 1. Introduction

Tobacco smoking is a major single cause of global cancer deaths and has been labelled as the biggest risk factor for premature deaths in industrialised countries such as the UK [1,2,3]. It is well known that tobacco smoking directly affects the cardiovascular system [4] through several mechanisms such as atherosclerosis, development of ischemic heart disease, and peripheral artery disease and when combined with other risk factors such as hyperlipidaemia, hypertension and obesity [5]. Nicotine and carbon monoxide, two major constituent chemicals in cigarettes, interfere with the ability of the cardiovascular system to function normally. Exposure to nicotine and carbon monoxide change the heart and blood vessels in ways that increase the risk of heart and cardiovascular disease [5,6]. Nicotine causes human blood vessels to constrict [6], which limits the amount of blood that flows to human organs. It is well known that the blood is the most important connective tissue in the human body that is primarily responsible for the distribution of nutrients and oxygen and the removal of carbon dioxide and metabolic waste products from organs. Any deviation in normal blood circulation, such as over or underflow may indicate a major systemic abnormality. Thus, as an effect of smoking, the constant constriction in the blood vessels results in stiff and non-elastic blood vessels. To compensate for this, the heart starts pumping more blood around the body resulting in its enlargement and increased heart rate [7]. This increased rate, an enlarged heart, and stiffer blood vessels make it harder to pump the blood and provide the body with the required amount of oxygen and nutrients [6]. These structural changes in the blood vessels and heart increase the risk of high blood pressure and cardiovascular disease, ultimately leading to higher mortality and morbidity among the smoking population bringing significant financial burdens on the National Health System and the economy [8,9].

Continuous monitoring of human health and activity using wireless wearable devices will be a key technology in the ubiquitous sensor network society for years to come. Recent advances in the development of the latest-generation of watch-size wearable devices based on ultra-compact semiconductor lasers have opened a new perspective for the implementation of compact blood flow monitoring sensor systems for personalised medicine [10,11]. This wearable device is based on laser Doppler flowmetry (LDF), which is closely related to the dynamic light scattering approach [12]. This technology is widely used for non-invasive measurements in living tissue optical parameters. These devices use a single-mode fibre coupled near-infrared (NIR) laser irradiation which is scattered and reflected from moving red blood cells (RBCs) [10]. Thereafter, these RBC movements generate the frequency-shifted scattering of the initial illumination which is detected by one or two photodetectors with the appropriate signal processing of both photocurrents (initial and reflected). This allows for the subsequent evaluation of the intensity of the blood perfusion. These data are then analysed with the Fourier approach of giving an estimation of the Doppler spectrum that means the LDF records are proportional to the RBCs’ velocity. Thus, LDF is used for functional diagnostics of the blood circulation system as well as significant diseases associated with cardiovascular disorders and their complications. Furthermore, this method allows evaluation of the oscillatory processes in the microcirculatory systems. Five rhythmic oscillations are isolated from LDF recordings with the help of wavelet analysis; endothelial (frequency interval 0.0095–0.02 Hz), neurogenic (0.02–0.06 Hz), myogenic (0.06–0.16 Hz), respiratory (0.16–0.4 Hz), and cardiac or pulse rhythm (0.4–1.6 Hz) [13]. Recent studies on these wearable LDF devices have shown the high synchronisation of blood flow rhythms in the contralateral limbs of healthy volunteers [11]. Additionally, it was reported that wearable devices can measure the age-related changes in blood perfusion for healthy participants [10]. Although there were quite a few numbers of articles available based on smoker’s blood microcirculation measured by LDF [14,15,16,17,18], no research was conducted in understanding the comparable modulation of the blood flow parameters in non-smoking and smoking volunteers using wearable LDF. Thus, this study demonstrated the feasibility of the wearable device in distinguishing cardiovascular parameters between non-smoking and smoking groups of volunteers. This preliminary research was necessary for planning more expensive and large-scale pre-clinical trials. Within the framework of the work, the strengths and weaknesses of the proposed LDF wearable approach were revealed. The applicability of the sensor for participants was assessed and estimation of the size of the participant groups were evaluated for obtaining sufficient statistical power of future studies.

## 2. Materials and Methods

### 2.1. Wearable Laser Doppler Flowmetry Monitor

**Figure 1:**
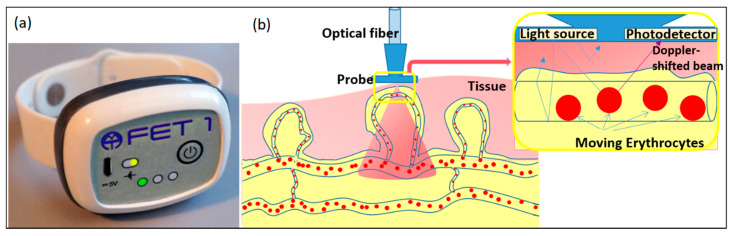
(**a**) Laser Doppler flowmetry (LDF) based wearable device, (**b**) schematic of the principle of LDF technique.

The experiments were performed with two wearable LDF monitors “FET-1” (Aston Medical Technology Ltd., http://www.amedtech.co.uk/) for recording the blood perfusion (Figure 1). These devices consisting of three identical channels for recording blood perfusion, skin temperature, and movements provide measurement at any desirable point of the human body. The system also comprises a wireless data acquisition module.

Every wearable sensor in the system uses a VCSEL chip (850 nm, 1.4 mW/3.5 mA, Philips, The Netherlands) as a single-mode laser source to implement fibre-free direct illumination of tissue. The fibre probe movements can cause high-frequency intensity fluctuations due to speckle movement. The intensity fluctuations can themselves produce an apparent Doppler shift [19,20] which will highly disturb the initial data acquisition creating faulty conclusions. Fibre-free solution and direct illumination of tissue by the laser diode make it possible to decrease these artefacts which are common in fibre-based LDF systems, as well as to avoid fibre coupling losses. To find a correlation between the changes in the registered blood perfusion and actual body movements the integral accelerometer has been embedded in the sensor.

The measurement channel of the devices uses two identical primary signal processing channels. This two-channel scheme provides a more accurate recording of the perfusion value (in particular, the recording of the Doppler shift). Mathematically, the recorded signal is represented as:

(1) $$PU=\int_{\omega_{1}}^{\omega_{2}}\omega S(U_{1}(t)-U_{2}(t))d\omega ,$$

where *PU* is perfusion index, $S(U_{1}(t)-U_{2}(t))$ is power spectral density of the difference signal from two photodetector channels, $U_{1}(t),U_{2}(t)$ is the signal from the photodetector converted to voltage.

The signal is also normalised to the constant component of the photocurrent. The radiation, scattered on moving red blood cells, and the back-reflected signal are received by two photodiodes. Next, the current is converted on the transimpedance amplifier, and the corresponding signal amplification is performed. The next stage of processing is low-pass filtering, constant component extraction, and high-pass filtering. Next, the variable component is normalised to a constant to avoid the influence of different levels of reflection under different optical conditions. The final component is a differential amplifier that protects the circuit from common-mode interference in the signal and reduces the impact of motion artifacts. In general, the presented wearable devices have an identical electronic circuit with stationary LDF devices [13,21,22] and allow the registration of the same signals.

### 2.2. Study Design

This pilot study was conducted in accordance with the principles set out in the Helsinki Declaration of 2013 by the World Medical Association and was approved by the human ethics committee of Aston University, Birmingham, UK (Ethics approval #1685). This pilot-study involved nine healthy volunteers without pre-existing cardiovascular and other chronic medical conditions who are divided into two groups: non-smokers and smokers. The average age of the non-smoker group was 34.2 ± 3.8 years and the smoker group was 38 ± 4.7 years. Two females and three males were included in the non-smoker group and smoker group consisted of 1 female and 3 males for this pilot study. None of the participants had a history of cardiovascular disease (CVD) or other illness and they were not under any medications. After being informed and explained the study design, every volunteer provided written consent and filled a questionnaire to detail their current health conditions. A short history for the volunteers including medication history, alcohol consumption for the last 24 hours, history of exercise (such as cycling, treadmill, jogging), and a detailed history of smoking were taken.

### 2.3. Testing Procedure

Blood perfusion parameters were collected in a sitting position, in a state of physical and mental rest (avoid reading, writing, and talking). The hand of the volunteers was placed on a table at the heart’s level and they were requested not to ingest any caffeine and alcohol-containing drinks at least one hour and twelve hours prior to allocated measurements time, respectively. The index of blood perfusion was recorded for 8 minutes, while the sensors were attached to the surface of the left middle fingertip and the topside of the left wrist without applying any pressure on the study area. The fingertip was chosen because it is rich in arteriolar venular anastomoses (AVA), whereas the wrist is known to contain less AVA. The measurement was taken twice a day: morning (around 11.00) and evening (around 18.00) for any 5 days in consecutive two weeks.

### 2.4. Data Proccesing and Analysis

**Figure 2:**
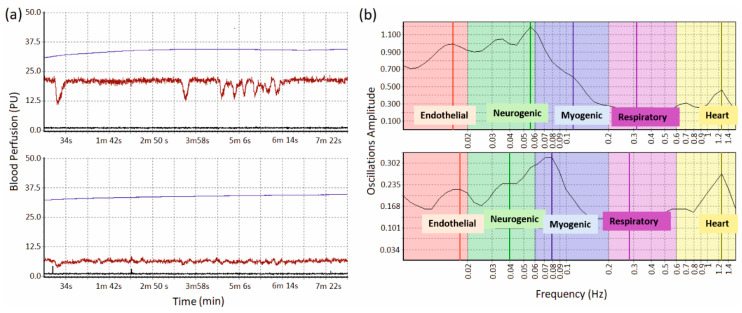
(**a**) Representative recordings of the blood perfusion (in brown), temperature (in blue) and accelerometric movement (in black) for left fingertip (upper) and left wrist (lower), (**b**) representative wavelet analysis for left fingertips (upper) and left wrist (lower).

Specialised software was developed to work with the system. This allowed for real-time control of the course of the experiment and analysis of the recorded parameters. Figure 2 depicts an example of the displayed parameters which show the raw data of blood perfusion, temperature, and the movement for the fingertip and wrist in Figure 2a. After acquiring the data, the oscillation rhythms of each measurement were analysed using the built-in module “wavelet analysis” [23] which is displayed in Figure 2b. This wavelet analysis determines the maximum amplitude of blood perfusion and corresponding data for each of the five oscillations mentioned in the previous section.

The Morlet wavelet transform was used for the frequency analysis of registered signals [24,25,26]. In short, the LDF signal was decomposed using a wavelet transform as:

(2) $$W(s,\tau )=\frac{1}{\sqrt{s}}\int_{-\infty }^{\infty }x(t)\psi^{*}\left(\frac{t-\tau }{s}\right)dt,$$

where *x*(*t*) is a target signal, *τ* is local time index, *s* is scaling factor, * means complex conjugation. The Morlet wavelet defined in the form

(3) $$\psi (t)=e^{2\pi it}e^{-t^{2}/\sigma }$$

was used with the decay parameter σ = 1. This wavelet allows one to ensure sufficient time-frequency resolution and is well localized in the time domain.

Figure 2 shows that the forearm region has smaller variations of the LDF signal, and spectral analysis shows pronounced peaks in the cardiac, myogenic, neurogenic, and endothelial ranges. We excluded respiratory fluctuations from further statistical analysis due to the lack of pronounced peaks, which may be due to recording signals in basal conditions without any functional tests [27,28].

Taking into account the relatively small sample sizes, nonparametric methods were used to confirm the reliability of differences in the results, namely the Mann–Whitney U-test. Values of *p* < 0.01 were considered significant. Based on the results of this pilot study, we have performed sample size estimations for minimisation of type two error in future studies:

(4) $$n=2SD^{2}\frac{{\left(Z_{\alpha /2}+Z_{\beta }\right)}^{2}}{d^{2}},$$

where is the SD is standard deviation; *Z_α_*_/2_ = 1.96 at type 1 error of 5%; *Z_β_* = 0.84 at 80% power; *d* is the difference between mean values [29].

## 3. Results

**Figure 3:**
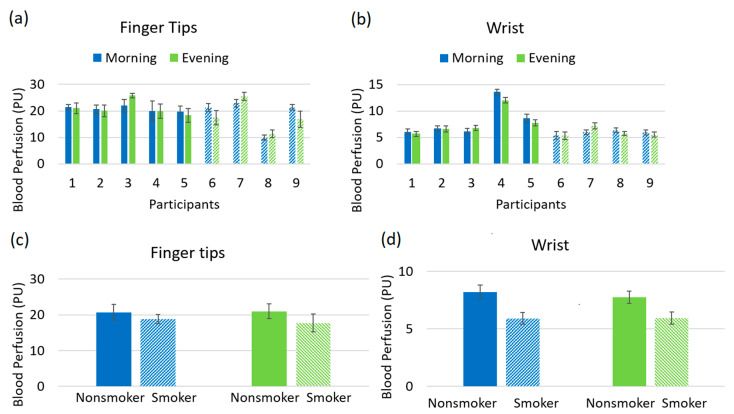
(**a**) Blood perfusion with standard deviation of left fingertip for every participant; (**b**) blood perfusion with standard deviation of left wrist for every participant; (**c**) averaged blood perfusion with standard deviation of fingertip for non-smoker and smoker, (**d**) averaged blood perfusion with standard deviation of wrist for non-smoker and smoker (solid bar denotes non-smoker and patterned bar denotes smoker).

Figure 3a, b shows the blood perfusion for all the individual participants including non-smokers and smokers at palmer skin of the left middle finger and left wrist, recorded in morning and evening sessions. It does not show any noticeable difference between non-smokers and smokers. After averaging for all non-smokers and smokers, the study reveals a higher level of perfusion in the non-smoker group as compared to the smoker group, showed in Figure 3c,d.

**Figure 4:**
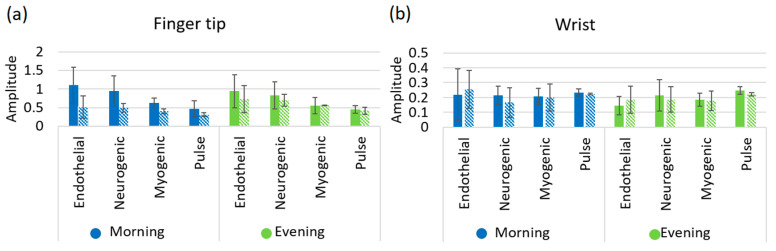
Maximum amplitude with standard deviation of the endothelial, neurogenic, myogenic and pulse mechanism for non-smoker and smoker at (**a**) fingertip, (**b**) wrist. Solid bar denotes non-smoker and patterned bar denotes smoker.

**Figure 5:**
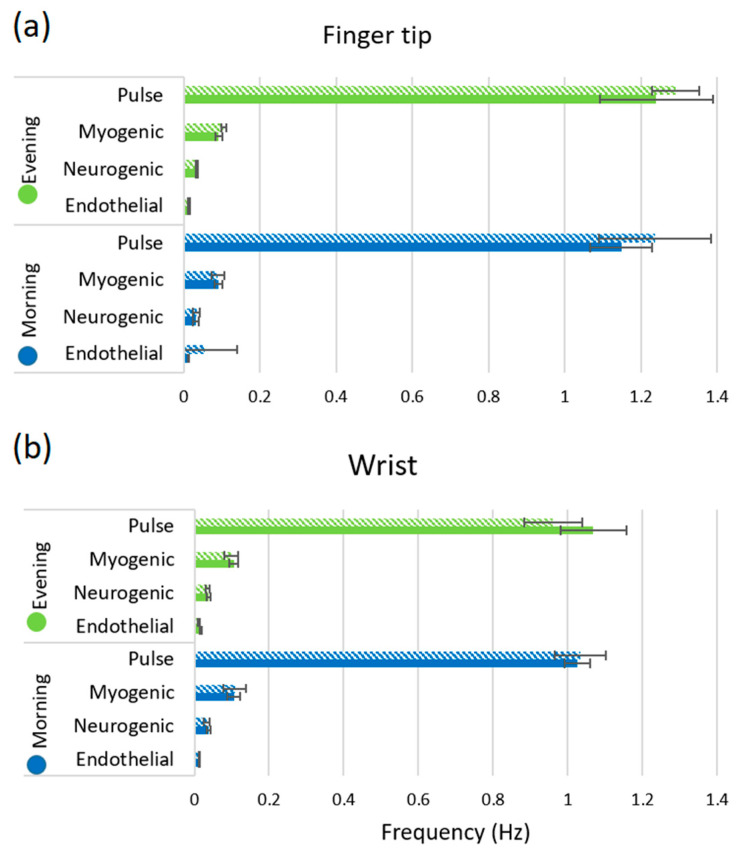
Maximum peak frequency with standard deviation of the endothelial, neurogenic, myogenic and pulse mechanism for non-smoker and smoker at (**a**) fingertip and (**b**) wrist. Solid bar denotes non-smoker and patterned bar denotes smoker.

The results depicted in Figure 4 and Figure 5 reveal the comparisons of the wavelet analysis parameters on the fingertip and wrist for both subject groups. The variations of the amplitude of the endothelial (A_e_), neurogenic (A_n_), myogenic (A_m_), and pulse (A_p_) are shown in Figure 4a,b for fingertip and wrist respectively. Amplitude measurements on the fingertip are consistently higher for the non-smoker group as compared to the smoker group for all oscillations at both morning and afternoon sessions. However, data received from the wrist does not show any conclusive variations between non-smoker and smoker groups. This is possibly due to the lower number of AVAs in the wrist area resulting in a lack of conclusive information. In addition, pulse amplitude data taken from both fingertip and wrist exhibits a decrease for the smoker group at both morning and afternoon sessions.

In Figure 5, the maximum peak frequencies are observed for endothelial, neurogenic, myogenic and pulse rhythms. The oscillation frequency varies for the smokers as compared to non-smokers, shown in Figure 5. It is clearly demonstrated that the pulse frequency is higher for the smoker group as compared to the non-smoker group at both morning and afternoon sessions for fingertip. Additionally, the wrist shows the same pattern for the morning session. This indicates that smoking raises blood pressure and increases the heart rate for smokers, which is confirmed in Figure 5a. For other oscillations, the frequency shows higher value for smoker than non-smoker’s fingertip at both morning and afternoon sessions, however, it is slightly different for wrist position.

## 4. Discussion

Here, we presented a pilot study to assess the feasibility of the wearable device in differentiating cardiovascular parameters between non-smoking and smoking groups of volunteers. Due to the small sample size, statistical analysis did not show significant differences, but the results show clear trends for differences in the measured and calculated parameters.

Lower blood perfusion for the smokers was observed compared to non-smokers in Figure 3c,d. This is probably due to the effect of nicotine, a major constituent of cigarette smoke. Nicotine constricts the blood vessels as well as those in the skin and coronary blood vessels. This vasoconstriction of the skin results in reduced cutaneous blood flow.

The amplitude of endothelial mechanism reduces for the smokers at fingertips, evidenced in Figure 4a. It is known that endothelial cells inside of the blood and heart vessels help to regulate blood clotting and vascular relaxation. These cells generally synthesize and release nitric oxide (NO) which dilate the blood vessels of the body. However, nicotine induces the chance to increase the endothelial dysfunction which is known to cause vasoconstricting substances and narrows the blood vessels [30]. As a consequence, the blood perfusion reduces for the smoker group, evidenced in Figure 3c,d. The plots for fingertip and wrist both show a decrease in amplitude of the neurogenic oscillation for smokers. This is an indicator of higher neurogenic resistance and possible decreased blood flow in the arterioles for the smoker group, which was noticed in the blood perfusion plot of Figure 3c [13]. The source of myogenic oscillation is representative of the spontaneous activity of smooth muscle cells that is associated with the regulation of blood pressure of the human body. Nicotine increases blood pressure for smokers and consequently increases the tension of the vascular wall resulting in the contraction of the vascular smooth muscle. The amplitude of myogenic oscillation decreases for smokers as shown in Figure 4a. Measurements from the wrist show a similar amplitude pattern in myogenic rhythm.

The pulse frequency was higher for the smokers as shown in Figure 5 because the cigarette smoking increases the cardiac work by stimulating the heart rate approximately 10–15 bpm and the blood pressure (acute increase 5–10 mm Hg) [31]. It was already discussed that nicotine constricts the blood vessels, but it can also increase coronary blood flow by increasing the cardiac output, causing subsequent flow-mediated dilation (FMD). It means that the heart needs to pump more due to constriction of the blood vessels that increases the cardiac output as well as heart rate which was evidenced in Figure 5. In addition, the prolonged exposure of carbon monoxide from a cigarette can increase carboxyhemoglobin concentration to as high as 10% for the heavy smoker and may induce anaemia as it binds more readily to haemoglobin than oxygen. As a result, it blocks oxygen-binding sites and impairs the release of the oxygen that is able to bind. Carbon monoxide induced hypoxemia enhances the chance of smoking-related thrombogenesis via increased blood viscosity as the body compensates it by increasing red blood cell mass.

Overall, the current study showed that the wearable device (FET-1) is capable of differentiating cardiovascular parameters between non-smokers and smokers. Our promising results demonstrate the robustness of both the data acquisition and the spectral analysis methods employed to characterise measured optical data. However, it is necessary to continue research in a broader way with a larger sample size to provide a clinical and statistically significant difference in blood perfusion parameters between non-smokers and smokers.

## 5. Study Limitations and Future Directions

It is shown that most of our results should be considered as preliminary estimates because the research has some limitations. The future work will focus on overcoming the limitations described below.

The number of studied subjects in this research was relatively small. High parameters divergence together with low difference leads to low robustness of the results. Spectral characteristics of LDF samples are characterised by high intra-subject variability. Our estimations have shown that the sample size should be estimated in at least 80 subjects in every group under consideration to minimize type II error.

This work had sufficient limitations due to the LDF sample length, which led to inaccurate interpretation on low frequency oscillations. For reliable statistics, one should ideally include 10 cycles for each of the frequencies under investigation. We had an 8 min recording, which was why the reliable results can be obtained only for frequencies higher than 0.02 Hz. For lower frequencies, the results presented demonstrate only the tendency towards to quantitative data.

## 6. Conclusions

The present pilot-study demonstrated that the applied wearable device (FET-1) is capable of differentiating cardiovascular parameters between non-smokers and smokers. It presents the results of blood perfusion measurements with the rhythmic oscillations using wearable VCSEL-based sensor system. Studies have shown a comparatively low blood flow for smoker volunteers than the non-smokers. This LDF based wearable sensor system has several advantages which open excellent prospects for a new type of experiments. Power-efficient VCSEL-based devices perform the long-term blood perfusion monitoring which is completely non-invasive. Experiments have shown that the introduction of wireless wearable devices for recording blood microcirculation is a convenient solution for use in medical diagnostics. The wearable implementation of LDF has a high potential in the field of monitoring cardiovascular diseases and is also of great interest for the diagnosis of other conditions associated with microvascular disorders. Portability and low sensitivity to motion artefacts make them mobile and suitable for home use. Furthermore, the sensor device demonstrates the spectral analysis of LDF signal using wavelet transformation to evaluate the regulatory mechanisms and to distinguish the difference between non-smokers and smokers. The presented pilot study of the wearable VCSEL-based LDF sensors provides early results that will need further validation with larger clinical studies.
